# Prosthetics and Techniques in Repair of Animal's Abdominal Wall

**DOI:** 10.1155/2016/9463186

**Published:** 2016-05-11

**Authors:** Gamal Karrouf, Adel Zaghloul, Mohamed Abou-Alsaud, Elie Barbour, Khaled Abouelnasr

**Affiliations:** ^1^Experimental Surgery Unit, King Fahd Medical Research Center, King Abdulaziz University, P.O. Box 80216, Jeddah 21589, Saudi Arabia; ^2^Department of Surgery, Anesthesiology and Radiology, Faculty of Veterinary Medicine, Mansoura University, Mansoura, Dakahlia 35516, Egypt; ^3^Biological Science Department, Faculty of Sciences, King Abdulaziz University, Jeddah, Saudi Arabia; ^4^Department of Animal and Veterinary Sciences, Faculty of Agriculture and Food Science, American University of Beirut, Beirut, Lebanon; ^5^Biochemistry Department, King Abdulaziz University, Jeddah, Saudi Arabia

## Abstract

The management of abdominal wall repair continues to present a challenging problem, especially in the repair of major defects. Many abdominal wall defects can be repaired by primary closure; however, if the defect is large and there is a tension on the closure of the wound, the use of prosthetic materials becomes indispensable. Many studies have been performed with various materials and implant techniques, without the comparison of their degrees of success, based on sound meta-analysis and/or inclusive epidemiologic studies. This review covered the effectiveness of recent advances in prosthetic materials and implant procedures used in repair of abdominal wall, based on biomechanical properties and economic aspects of reconstructed large abdominal wall defects and hernias in animals. The presented results in this review helped to reach treatment algorithms that could maximize outcomes and minimize morbidity.

## 1. Introduction

The abdomen is a very delicate part of the body of animals. It is subjected to trauma and clinical disorders. Hernias are quite common in both young and mature animals. In massive abdominal wall defects, the use of graft becomes mandatory to achieve desirable results. Different techniques have been performed to overcome this challenge [1].

Many abdominal wall defects and hernias can be repaired by a primary closure while massive defects, including irreducible hernia, need special attention, since they cannot be treated by simple methods of reduction. This type of hernia requires surgical procedures to rectify the defect by the use of graft [2].

The concept of tension-free herniorrhaphy is a potential resolution of the controversies that have surrounded the subject of hernia surgery for more than a century. Certain hernial cases involve massive loss of abdominal muscles which cannot be repaired by simple suturing of opposing muscles; thus, grafting becomes the only option for their repair [3].

The current standard of practice is to repair most defects using permanent synthetic mesh material. The mesh is known to augment the strength of the weakened abdominal wall fascia and to enable the hernia repair under a tension-free condition. However, this surgical procedure could be associated with a risk of infection, fistula formation, and possibility of presence of chronic abdominal wall pain. In avoidance of these postoperative injuries, surgeons are directing their efforts towards the use of xenogenic and allogenic materials for the repair of abdominal wall defects [4].

The management of complex abdominal wall defects is challenging and remains a major problem for patients and surgeons, due to insufficient availability of an autogenous tissue for adequate abdominal wall closure, and often requires an individualized strategy with additional measures to minimize recurrence [5, 6]. The use of biomaterial for the repair of abdominal wall defects has gained an increasing recognition in achieving a tension-free repair, resulting in a significant reduction of postoperative pain, shortening the recovery period, and the frequency of recurrence [7].

## 2. Prosthetic Materials in Repair of Abdominal Defects

The development of prosthetic materials for the repair of abdominal wall defects has evolved and progressed during the past several years, with the ultimate goal of discovering the “ideal prosthesis.” The classic polyester, polypropylene, and expanded polytetrafluoroethylene have been replaced by materials of natural origin, mainly from animal sources. These implants, named as “biomeshes,” are primarily composed of collagen, with their ability to regenerate new tissue in the human recipient, while the biomeshes are undergoing a progressive degradation [8].

The ideal biomaterial for abdominal wall repair should posses adequate strength and no hypersensitivity reaction, be durable and pliable, have grainy texture to grip the peritoneum and prevent slippage, resist infection, and be reactive enough to induce a rapid fibroblastic reaction and biocompatibility to facilitate tissue in growth, which may help long term maintenance of mechanical strength [5]. In addition, such materials should have the capacity of tolerance by the living tissue, avoiding rejection, while they are absorbed naturally by the biological processes of the body, or staying intact or partially intact, as a permanent part of the surrounding tissue [9].

### 2.1. Synthetic Prosthetic Materials

More than 80 types of synthetic mesh were used in repair of abdominal wall and fascia. The synthetic materials are divided into nonabsorbable and absorbable mesh. The nonabsorbable mesh includes stainless steel, tantalum, Teflon, Orlon, silicon, monofilament or polyfilament nylon, polypropylene, polytetrafluoroethylene, polyethylene, Dacron fabric, fiberglass, polyester, and Marlex and Mersilene mesh; however, the absorbable mesh includes fewer materials, namely, polyglycolic acid, polyglactin 910, and Bulgarian antimicrobial polyamide [7].

The most important necessary characteristic for a successful mesh repair is the strength of the tissue incorporation. However, this characteristic could lead to a tendency towards adhesion formation. For this reason, the development of the optimal material for mesh hernia repair was essentially a balancing act between the strength of tissue incorporation and adhesion formation [7].

The utilization of polypropylene mesh, discovered 40 years ago, became increasingly popular, due to its documented biocompatibility. This synthetic mesh achieved a tension-free environment at its site, resulting in significant reduction of postoperative pain, shortening of the recovery period, and reduction in frequency of postoperative reoccurrence. However, the disadvantages of this mesh lie in its inelastic nature and the high cost. Alternatively, some surgeons choose to use biologic materials that meet the structural requirements and safety [7, 10].

The easy handling of polypropylene mesh helped in their frequent application for repair of abdominal wall defects in horses, ponies, cattle, and dogs. The cut edges of this mesh resist fraying and tissue granulation, helping capillaries to grow through the interstices of the mesh and strengthening its incorporation. The negative side is the observation of a tendency towards an adhesion formation [11]. The comparison of polypropylene versus polytetrafluoroethylene patches showed that the first was associated with a significantly lower incidence of recurrent herniation, rapid fibrinous fixation to the host tissue during short time, and retaining of its original square shape; however, the expanded polytetrafluoroethylene (ePTFE) showed an incomplete fibrinous fixation during the same period of time, manifested by wrinkling and distortion and associated with a low rate of adhesion formation [7, 12].

The partially absorbable reticular prostheses are now available on the market (Vypro II and Ultrapro), inducing an acceptable tissue growth, providing considerable strength to the repair zone, while absorption of some of the components of the mesh occurs in the host. This absorption reduces the amount of foreign material persisting in the host, without compromising the biomechanical resistance of these meshes. In addition, these partially absorbable meshes have the possible benefit of flexibility and more compliance through a longer implant period [8].

Using Sepracoat, a viscous solution composed of 0.4% hyaluronic acid in phosphate buffered saline and icodextrin, decreases the incidence of bowel or omental adhesion to the mesh, by the mechanism of coating the implant with a temporary protective layer that is completely resorbed from the abdomen within five days [13].

Parietex composite mesh is a nonabsorbable polyester mesh coated with an absorbable and hydrophilic film on the visceral side, which gets absorbed within 3 weeks, and a new peritoneal covering will be formed over the mesh with significant protection against bowel adhesion [14]. Unfortunately, the Parietex composite mesh is more easily infected than other meshes, resulting in an augmented inflammatory response [15].

### 2.2. Natural Prosthetic Materials

Many clinical complications could follow the abdominal wall repair by synthetic materials including wound infection, bowel fistula, erosion into abdominal viscera, increased recurrence rate, repair failure, and mesh extrusion. In addition, the high cost associated with synthetic material implants initiated the search for safe and cheap biodegradable material that have enough strength to support the abdominal wall during the healing process, with the ability of replacement by the recipient fibrous tissue before its complete degradation. Recently new biomaterials derived from biological material of a collagen nature including cadaveric fascia lata [16], tunica vaginalis [17], bovine pericardium [18], and collagen based material derived from porcine small intestine submucosa [19] had been tested for the repair of abdominal wall defect.

The repair of abdominal wall defects by biological biomaterials has better advantages over the synthetic prosthetic materials, due to their minimal adhesion formation, providing better framework for fibroblast proliferation, neovascularization, and building of multidirectional fibrous structure. These results help in better suture retention, while the material is absorbed and replaced by the host tissue [20].

Despite the acceptable results obtained by biological prostheses, several clinical complications are reported in xenograft biologic mesh implantation including evisceration, disintegration, poor mesh integration, and infection or seroma [21, 22].

A recently published systematic review stated that wound infection and seroma formation are the most common postoperative complications related to biological prostheses implantation [23].

A resemblance of collagen-derived biological prostheses is synthesized from two copolymers and is known as Bio-A. This material can undergo a progressive biodegradation through hydrolytic and enzymatic processes that do not leave any permanent residues in the host. Tuto is a non-cross-linked prosthesis created from bovine pericardium that has been used in the repair of multiple structures [24]. Another experimental study related to total abdominal defects in rats revealed that the bovine pericardium and polytetrafluoroethylene elicit a minimal foreign-body reaction and provide adequate mechanical resistance as early as two weeks after implantation. In addition, both collagen meshes elicited a similar macrophage response that decreased over time [25]. Clinical studies on contaminated tissues demonstrated that the cellular bovine pericardium provides a suitable repair for major abdominal defects [26, 27].

Actually, the bovine pericardium was used as a biomaterial for manufacturing various bioprostheses, due to their inherent strength and biocompatibility. It was also used after treatment with different cross-linking methods, for the construction of bioprosthetic heart valves, and for repair of several soft tissue deficiencies, such as cardiac patches, and vascular stent related to cardiovascular and lung surgery [28, 29].

The parietal tunica vaginalis is a serous sac that is formed by an outpouch of the parietal peritoneum of the abdominal cavity during testicular descent. The inner surface of the parietal tunica vaginalis is separated from the visceral tunica vaginalis by the vaginal cavity and the outer surface of the parietal tunica vaginalis is slightly fused to the scrotal fascia, allowing for its easy separation [30]. Bovine tunica vaginalis seems to be a suitable biomaterial for repair of abdominal wall defect. It is available, highly incorporative to host tissue, inexpensive, tolerable by the host immune system, and not prone to fragmentation or infection [17]. It does not stimulate the formation of any adhesion with the underlying viscera and induces a minor inflammatory reaction compared to pericardium, when grafted for repair of abdominal wall defect in sheep. The technique of implantation of the prosthetic materials is thought to play an important role in hernia recurrence [31, 32].

The experimental study [33] revealed that the bovine tunica vaginalis implant was more advantageous than the bovine pericardium. Its success could be due to its derivation from the peritoneum, which is the serous membrane originally lining the wall of the abdominal cavity. Thus, it has the properties of the natural abdominal wall, with low incidence of rejection, which could be due to its poor supply of blood vessels. It is worth noting that the bovine tunica vaginalis was not completely resorbed at the 30th day after implantation, which might be attributed to its high density of fibrocollagen compared to that of the bovine pericardium. There was no occurrence of host muscle-cell migration or lack of skeletal muscle tissue development in the constructed area [33].

Iqbal et al. and Franz et al. [2, 34] revealed that mesh herniorrhaphy has an edge over jejunal grafting in some respects. The mesh herniorrhaphy is an easier technique compared to the complex jejunal grafting. The polypropylene is quite available, rendering it as a sole method in emergency incisional hernia, when the animal life is at risk. Polypropylene mesh graft is known to provide a 100% healing of the abdominal wall defect. On the contrary, autografting creates more stress on the animal, resulting in fluid loss, and higher chances of death. Moreover, the mesh herniorrhaphy is less time consuming and safer.

The biological biomaterials, in their naturally occurring form, tend to deteriorate over time, hence, achieving safe long-term storage, minimizing the immunogenicity of the graft, and stabilizing the tissue against rapid enzymatic and chemical degradation. It is generally necessary to alter the physiochemical state of the biomaterials by synthetic chemicals such as formaldehyde, dialdehyde, starch, and glutaraldehyde [35, 36].

Glutaraldehyde cross-linking is proven to minimize the immunogenicity of the graft, stabilizing the tissue against rapid enzymatic and chemical degradation. However, its application has also been associated with several problems including altered mechanical properties and early mechanical failure, calcification, cytotoxicity, and incomplete suppression of immunological recognition [37].

Glycerin was able to preserve the bovine pericardium grafts in abdominal wall repair of rats, showing a reduced amount of abdominal viscera adhesion and more implant incorporation with the host tissue in comparison to that of the polyester mesh [38]. Glycerol pretreatment seems to delay the implant biodegradation and replacement by host tissue compared with freeze-drying. This pretreatment has a similar effect to that performed by glutaraldehyde, resulting in delay of biodegradation and resorption of biological implants. However, due to cytotoxic and calcification effect attributed to glutaraldehyde pretreatment, the glycerol preservation is still preferred [35, 39]. These workers found that glycerolized tunica vaginalis has a higher histocompatibility and safety in repairing of abdominal wall defect than synthetic meshes, with more advantages of glycerolized tunica vaginalis, due to its simple procedure, lower cost, and pronounced safety.

The formation of intra-abdominal adhesion is one of the clinical problems associated with the use of prosthetic materials, leading to fistula, and that might require reoperation and removal of the patch. Processed bovine tunica vaginalis showed an apparent advantage, in which its placement in direct contact with the underlying viscera does not result in an intra-abdominal adhesion. However, adhesion was restricted between the peritoneal side of the biomaterial and the greater omentum [17].

The ideal concentration of glutaraldehyde to be used in the preservation of the material was optimized at 0.5%, while higher concentrations of 2.5, 5, and 10% showed negative effects on the pericardial tensile strength and elongation [40]. Glycerol was able to preserve the bovine pericardium, showing higher histocompatibility with the host tissue, an absence of postoperative complication, associated with a slight adhesion between implanted grafts and some visceral organs [18].

Previous reported study by Coito and Kupiec-Weglinski [41] revealed that glutaraldehyde treatment does not completely eliminate the immune response to allograft and xenografts and can induce calcification. This indicates that glycerol preservation technique is preferable to glutaraldehyde. In addition, glycerol preservation is simple and inexpensive, while glutaraldehyde preservation is complex and costly [39]. These workers added that the use of glutaraldehyde for the preservation of bovine pericardium originated from its success in cardiac surgery for maintaining its flexibility. However, the thoracic and abdominal wall reconstruction does not give flexibility too much importance.

The glycerol-preserved bovine pericardium and tunica vaginalis implants were gradually resorbed and replaced with the recipient generated tissue at different rates. Researchers found that glycerol pretreatment seems to delay the implant biodegradation and replacement by host tissue compared with other types of preservation such as the lyophilized, irradiated, and freeze-drying methods [35, 39].

Although freeze-drying and gamma irradiation were the techniques widely used in tissue banking for preservation and sterilization of tissue graft, respectively, the effects of these techniques on biomechanical properties of bovine pericardium were poorly known [39]. Lyophilization is a dual stage process that consists of rapidly cooling the liquid material to its solid form and subsequent drying under low pressure and temperature, leading to preservation of bioprostheses [35]. This process can be combined with gamma irradiation prior to surgical application without any significant effects on the biomechanical properties of the bovine pericardium [39, 42].

The effectiveness of lyophilized bovine pericardium and tunica vaginalis parietalis in repairing of abdominal wall defect in rabbit model is investigated by Ayele et al., [43]. The postmortem examination revealed that all of the implanted materials retained their original rectangular shape and were incorporated with the recipient abdominal wall. In addition, the prolene mesh was encapsulated with dense fibrous tissue, showing wrinkling of the mesh, while the bovine pericardium and tunica vaginalis were coated with connective tissue layer and new peritoneum with formation of neovascularization.

The formation of neoperitoneum, acting as a barrier leading to a decrease in adhesion formation, was observed in rabbits implanted with tunica vaginalis (GTV) and bovine pericardium (GBP). Matthews et al. [44] and Ayele et al. [43] proved that the processed bovine pericardium and tunica vaginalis can be placed in direct contact with underlying viscera without stimulating an intra-abdominal adhesion. However, adhesion was formed between the peritoneal side of the implant and the greater omentum, which was attributed to formation of a lesion caused by abrasion, ischemia, and foreign body.

The use of polypropylene led to extensive visceral adhesions, leading to intestinal fistula. This is due mainly to the mesh higher ability for incorporation with the surrounding tissue of the abdominal wall. This adhesion was more intense in rabbits implanted with prolene mesh than in those treated with GBP and GTV. Actually, the adhesion was graded with 1, 2, and 3 levels that required aggressive blunt dissection [11].

## 3. Techniques of Implantations

The most effective positioning of the prosthesis is also a subject of debate. Three techniques are known for implantation of biomaterials to bridge an abdominal wall defect. These techniques include “inlay” sewing the mesh into the fascial defect, “onlay” (a superficial technique) in which the fascial suture is reinforced by placing a mesh over it, and the “underlay” positioning of the mesh in the retromuscular space, posterior to the rectus abdominis muscle and in direct contact with viscera after omentalization [31, 45].

The “inlay” technique is preferred in the repair of large ventral incisional hernia, in which the mesh is sewn to the margins of the defect by simple continuous suture, interrupted only at the corners. This is described as the simplest form of repair, but it suffered from high relapse rates, since no broad mesh contact between the fascia and the prosthetic material is established [45].

Although the inlay technique is the simplest form of repair, it has a disadvantage of lacking fixation of the implant by intra-abdominal pressure, due to minimal surface area of contact between the implant and the adjacent tissue, leading to higher frequency of relapse.

The “onlay” technique of implantation has the advantages of easiness in implanting the mesh, the easiness in removal of infected stitches, and the decreased strain on the suture line due to the spread of the tension across the mesh. However, it has minor ability to relieve tension and may cause local discomfort and erosions of mesh through the subcutaneous tissue and skin [46]. The “onlay” hernia repair has several disadvantages including tenderness of the abdominal wall, seroma formation, and highest rate of surgical site infection as well as mesh displacement from the intra-abdominal pressure [7].

**Figure 1:**
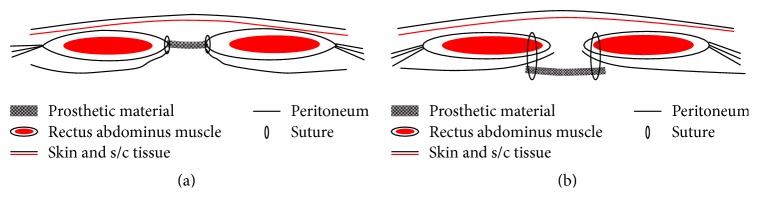
The techniques used for implantation of prosthetic materials [47]. (a) Inlay technique. (b) Underlay technique.

The “underlay” retromuscular position has the advantage of excellent incorporation into the abdominal wall with sufficient protection of the viscera, although an extensive tissue dissection is required. The “underlay” technique was considered the best method because of its relatively low hernia recurrence rates. Intraperitoneal placement of polytetrafluoroethylene mesh has several advantages over other techniques, including minimal dissection, providing better fixation and possibly a decreased risk of infection. The disadvantage of intraperitoneal placement of mesh grafts is the contact of the prosthesis with viscera, which could lead to inflammatory response, resulting in intra-abdominal adhesion, for which omental interpositioning as a physical barrier is recommended [48]. This technique needs a covering of the mesh with a fascial flap derived from the hernial sac, to provide an additional strength to the wound and to reduce the serous effusions as seen in Figure 1 [47]. In addition, it was stressed that a belly bandage should be applied to counteract seroma formation and to prevent soiling of the incision [39].

Hernia recurrence was less common in dogs implanted with “underlay” technique in comparison to those implanted with “inlay” technique. This may be attributed to the minimum contact of the prosthetic material to the host tissue; in addition, this lower frequency of recurrence could be due to interrupted sutures used for fixation of the implant in the “underlay” technique that can provide a multiple point nontension fixation, which help in distributing the stress over the mesh and reducing crimping and bulging of the mesh [1]. Thus, the “underlay” technique is currently considered the best method, because of its relatively low hernia recurrence rates, due to the positioning of the implant behind the rectus muscles where the force of abdominal pressure holds the prosthesis against the deep surface of the abdominal muscle wall [45, 49].

Higher degree of adhesion formation was encountered in animals implanted with “inlay” technique without omentalization, compared to the “underlay” technique with omentalization, which could be attributed to the direct contact between the mesh and the abdominal viscera which lead to inflammatory response to the prosthesis, giving rise to intra-abdominal adhesion [48]. These workers recommended the interpositioning of the omentum to act as a physical barrier between the implant and the viscera.

## 4. Tensiometric Evaluation

**Figure 2:**
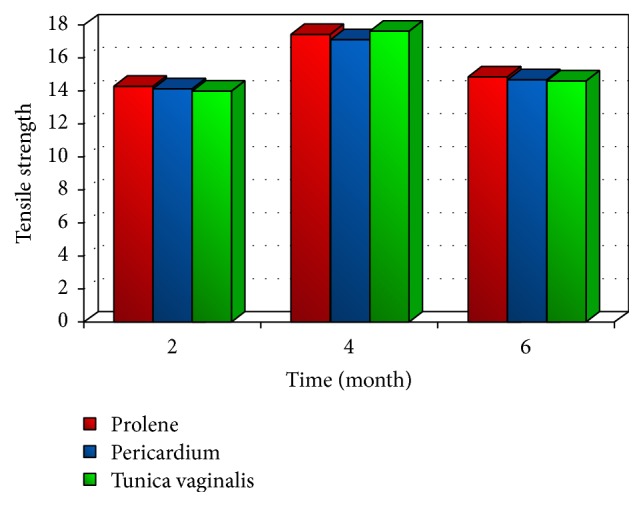
Tensile strength of prolene, pericardium, and tunica vaginalis at different times [33].

**Figure 3:**
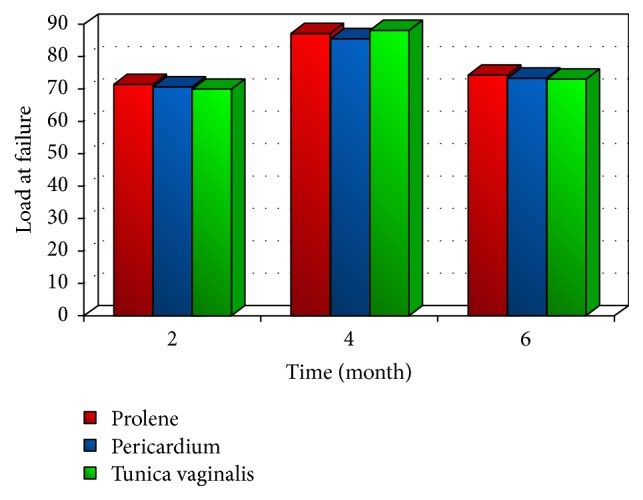
Load at failure for prolene, pericardium, and tunica vaginalis at different times [33].

The tensiometric evaluation is used to compare the success of implant by different materials in the repair of abdominal walls. Testing of the biomechanical properties revealed no significant difference in load at failure and tensile strength between prolene mesh, pericardium (GBP), and tunica vaginalis (GTV) at different times during a euthanasia period. However, the extension percent showed significant differences between the three materials at all time intervals. All tissue failures detected by tensiometric evaluation occurred either at the implant-muscle interface or at the muscle on periphery of the implant (Figures 2 and 3) [38]. This type of failure indicates a lower tensile strength of the muscle and fascia compared to that of the repair site [47, 50].

The differences among overall mean values of load at failure and healing tensile strength of prolene mesh, GBP, and GTV implants were not statistically significant at each specified time interval, due to the fact that all the implanted materials were sufficiently strong in maintaining the abdominal wall integrity [16, 39]. The load at failure and the tensile strength of healing abdominal wall, repaired with different prosthetic materials, increased gradually with time and peaked at 4 months after implantation, due to sufficient deposition of fibrous tissue and the decline in inflammatory response [39].

Tensiometric evaluation, including parameters of maximum load at break, healing tensile strength, and Young's modulus of elasticity of abdominal wall, revealed an insignificant difference among overall mean values of measured parameters resulting from tunica vaginalis and ePTFE mesh implants [51]. The elicited histologic response to the different types of implants including incorporation, encapsulation, mixed incorporation, and encapsulation and resorption did not affect the tensile strength of the xenograft or the synthetic mesh repair [52].

The bovine pericardium preserved in 0.5% glutaraldehyde showed significantly higher tensile strength compared to that preserved in 2.5, 5, and 10% glutaraldehyde. However, the percentage of elongation was significantly lower than that obtained in mesh preserved in 1, 2.5, and 5% glutaraldehyde [53]. The tensile strength values of the implanted bovine pericardium decreased in the first month after the implantation, and then they increased again after a decline in the host inflammatory reactions [54].

Freeze-drying treatment of the bovine pericardium mesh caused significant decrease in its thickness. However, this treatment did not affect significantly its tensile strength, Young's modulus (stiffness), and elongation rate. The gamma irradiation treatment caused a significant decrease in these properties, with no significant effect on its thickness [51].

The mesh-repaired wound had a markedly greater ability to elongate before disruption compared with suture-repaired wound. This elongation was highly statistically significant at all postoperative time points, reaching double that obtained by the suture-repaired wounds. It was observed that, for every 1 mm increase in wound elongation, the risk of mechanical disruption decreased by 22%, concluding that the mesh-repaired wounds have a significant mechanical advantage over the suture-repaired ones [55].

## 5. Histopathological Findings

The histopathologic findings were used to evaluate the success of protocols used in repaired abdominal walls. The gross inflammatory changes were shown to disappear gradually within the first 5 days after the operation on sheep that were grafted with tunica vaginalis; however, it took around 2 weeks for these inflammatory reactions to disappear in animals grafted with pericardium and 20 days in animals implanted with prolene. The differences in persistence of inflammatory reactions could be due to differences in immunogenicity of these meshes [32, 39].

Microscopically, the prolene mesh, appearing without changes in its fibers, was surrounded with a delicate layer of fibrous tissue and collagen fibers that became denser by time. This dense fibrous capsule is most likely responsible for the firm incorporation between the mesh and the host tissue but could be the cause of the observed wrinkling in this type of grafted mesh [56]. In the GBP and GTV implants, the dense collagen fibers of the implant were still present at two months but gradually resorbed and replaced with a delicate, immature, and unorganized fibrous tissue at four months. At six months after operation, the amount of collagen fibers increased and infiltrated throughout the biomaterials in a well-defined and organized form [39].

Mild calcification was observed at six months in dogs implanted with prolene mesh, while in animals repaired with GTV and GBP the calcification did not exist at all times. This observation indicated the histocompatibility differences in these materials to the recipient tissue. The studies gave an advantage to glycerol preservation over the other types of preservatives, such as glutaraldehyde that resulted in cytotoxic effect and calcification formation [32]. Myoblasts have been observed at the junction area between the host tissue and the GBP and GTV implants and then changed into mature and well-defined muscle fibers and extended inward into the biomaterial. These myoblasts were possibly derived from the mesenchymal cells or formed as an outgrowth of muscle fibers on either side of the injury [17].

## 6. Conclusions

The management of complex abdominal wall defects remains challenging. The current standard of practice is to repair most defects using prosthetic material. Appropriate judgment is required to optimize surgical outcomes in many complex cases. Prosthetic materials are shown to augment the strength of the weakened abdominal wall fascia and enables the hernia repair to be performed in a tension-free manner. However, these prostheses are still inadequate with respect to their precise time points of biodegradation, which fundamentally affects the repair process. One of the areas for improvement and research is the control of the prosthetic degradation times. Additional clinical studies and postoperation epidemiologies are required to better understand the long-term efficacy and limitations of these materials and to be able to compare them properly. Bovine pericardium and tunica vaginalis were biocompatible tissue replacers providing a good alternative to prolene mesh in repairing the abdominal wall defects and hernias in dogs, calves, foal, and sheep. In addition, these biocompatible tissue replacers are available at lower cost. Further research needs to be done in relation to regeneration of skeletal muscle tissue, using tissue engineering technologies.
